# A green separation mode of synephrine from *Citrus aurantium* L. (Rutaceae) by nanofiltration technology

**DOI:** 10.1002/fsn3.1265

**Published:** 2019-11-13

**Authors:** Cunyu Li, Yun Ma, Jiamei Gu, Xinglei Zhi, Hemin Li, Guoping Peng

**Affiliations:** ^1^ College of Pharmacy Nanjing University of Chinese Medicine Nanjing China; ^2^ Jiangsu Collaborative Innovation Center of Chinese Medicinal Resources Industrialization Nanjing China; ^3^ The Fourth People's Hospital of Taizhou City Taizhou China

**Keywords:** alkaloids, lipid‐lowering activity, mass transfer process, nanofiltration, synephrine

## Abstract

Thermal breakage of alkaloid ingredients was a common problem to which attention should be paid in the application of fruit ingredients separation. In this study, the mathematical models were established to predict the rejection of synephrine from *Citrus aurantium* L. (Rutaceae). The experiment showed that there was a linear relationship between operation pressure and membrane flux. Meanwhile, under the influence of solution–diffusion effect and the charge effect, the mass transfer coefficient was power functioned with initial concentration. The mathematical model showed that the predicted rejections of synephrine from *Citrus aurantium* extract were well approximate to real ones, and the lipid‐lowering active ingredient had effectively enriched. The predicted model of nanofiltration separation has a preferable applicability to synephrine and provides references for nanofiltration separation, especially for raw food materials with synephrine.

## Graphical abstract

Thermal breakage of alkaloids ingredients was a common problem to which attention should be paid in the application of fruit ingredient separation. In this study, the mathematical models were based on the operating pressure and initial concentration to predict the rejection of synephrine. Moreover, the concentration of synephrine was effectively increased and the lipid‐lowering activity was also enhanced by using NF technology. The predicted model of nanofiltration separation has a preferable applicability to synephrine and provides references for nanofiltration separation, especially for raw food materials with synephrine.

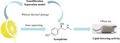

## 1 INTRODUCTION

Nanofiltration (NF) is a type of pressure‐driven membrane separation technology, whose molecular weight cutoffs (MWCO) ranges from 100 to 1,000 Da. The NF process, which has the advantages of low energy consumption, nonpollution, and high permeability, is growing as a hopeful technology for the process of purification and separation of active ingredients without heat effect (Imbierowicz, Troszkiewicz, & Piotrowska, 2015; Khanal, Howard, & Prior, 2010). NF application in food ingredients processing is still in its infancy. Due to the complicated structures of food composition, it is difficult to explain its separation behaviors and predict its separation rules depending on classical separation models, including the solution–diffusion model (SDM), pore flow model (PFM), and Donnan–steric partitioning pore model (DSPM; Hidalgo et al., 2013; Kumar, Hariharan, Mayya, & Han, 2013; Vezzani & Bandini, 2002).

Synephrine, a typical alkaloid in *Citrus aurantium* L. (Rutaceae), has an active effect on weight‐losing. Some studies suggest that it can cause the increasing arterial blood pressure and left ventricular pressure by strengthening cardiac output and raising the total peripheral vascular resistance (Koshani, Ziaee, Niakousari, & Golmakani, 2014; Rossato, Costa, Limberger, Bastos, & Remião, 2011). Synephrine has the empirical formula C_9_H_13_NO_2_, and its molecular weight is 167.21, and the stability of this component can be affected by prolonged heat and light (Pellati & Benvenuti, 2007).

Because of the alkalinity and high polarity, high‐performance liquid chromatography (HPLC), high‐speed counter‐current chromatography (HSCCC), and pH‐zone‐refining counter‐current chromatography (CCC) were used in separating synephrine (Pellati, Cannazza, & Benvenuti, 2010; Zhang et al., 2016), but the separation efficiency hardly be achieved, such as separation equipment, environmental pollution, and structures heating transformation. NF is a potential technology for efficient reservation of foods and medicine without heat effect, but the NF separation behaviors of synephrine are unclear. Based on the solution–diffusion and Donnan effect (Yaroshchuk & Bruening, 2017), a commercial NF membrane was used to clarify the relationship between membrane transport mechanisms and molecular state, and construct mass transfer model to predict separation behaviors. Given today's green separation demand over the world, NF can effectively improve separation efficiency and reduce environmental pollution.

## 2 MATERIALS AND METHODS

### 2.1 Materials

*Citrus aurantium* were obtained from a local market, which were from Zhangshu (Jiangxi, China). Synephrine extract (>98.0%) was purchased from Nanjing Zelang Biological Technology Co., Ltd. Synephrine reference substance (>98.0%) was obtained from the National Institute for the Control of Pharmaceutical and Biological Products (Beijing, China). HPLC grade methanol was purchased from Merck. All the other chemicals used in the experiments were of analytical grade, and deionized water was used.

NF membrane was carried out by using polyamide membrane with negative charge, molecular weight cutoff of 300 Da, a filtration area of 0.30 m^2^, max cross‐flow operation pressure of 3.0 MPa, MgSO_4_ minimum rejection of 99.0%, and permeation flux of 42.5 L/(m^2^ hr; Tuozhu Corporation). The apparatus consisted of a NF membrane, one peristaltic pump (Model TNZ‐1, Tuozhu Corporation) for pressure and recirculation, and a digital pressure gauge (Mettler Toledo) for the measurement of operating pressure. KH‐250B ultrasonic cleaner was used to accelerate the dissolution rate of synephrine extract in deionized water (Hechuang Ultrasonic Instrument). The pH values of synephrine were measured by PB‐10 pH Meter (Sartorius). Samples were determined by Agilent 1100 HPLC with VWD detector (Agilent Technologies).

Sprague Dawley (SD) adult male rats were purchased from Nanjing jiangning district Qinglongshan animal breeding center (number of animal license SYXK 2017–0011). Standard feed was provided by Animal Experiment Center, Nanjing University of Chinese Medicine, and the high‐fat feed was composed of 10% lard, 10% egg yolk powder, and 80% standard feed. Total cholesterol (TC) test kit, triglyceride (TG) test kit, high‐density lipoprotein cholesterol (HDL‐C) test kit, and low‐density lipoprotein cholesterol (LDL‐C) test kit were purchased from Nanjing Mallbio biotechnology Co., LTD.

### 2.2 Sample preparation

*Citrus aurantium* extract solution: The *Citrus aurantium* were washed with purified water, and fruit branches were cut off. The *Citrus aurantium* was processed in a commercial juicer to yield the natural juice. The natural juice was pretreated by a 0.45‐μm microfiltration membrane and kept at 4–7°C to prevent damage or degradation.

Synephrine reference solution: 0.0102 g synephrine reference was added to 50% methanol water, which was used as synephrine reference solution at a concentration of 1.02 mg/ml.

Synephrine extract solution: Synephrine extract was dissolved in deionized water with the concentration of 200 μg/ml.

### 2.3 pK_a_ measurement

The pK_a_ value of synephrine was determined to analyze the existence of solute so that it can adjust the existence of solute, and based on this, the effect on mass transfer process can be investigated. Since synephrine molecular structure contains imide group, the pK_a_ measurement of synephrine was according to the determination of monobasic weak alkali. The pH value was measured by a glass pH electrode at room temperature. First, the 20 ml precise amount of series of concentrations of synephrine extract should be placed in a clean dry beaker especially, according to hydrochloric acid standard titration. Then, the pH value was recorded once when 1.0 ml hydrochloric acid of the concentration of 0.1003 mol/L was added into the extract. After that, draw a curve with the value of pH and the data of hydrochloric acid volume (*V*). When the slope of this curve was close to 1.0, the amount of hydrochloric acid was changed to 0.2 ml each to record the pH value. Finally, the titration process can be ended until the slope of the curve went to infinity. By repeating the titration operation for three times, the pK_a_ value can be calculated according to Equation (1).

(1) $$\text{pKa}=\text{pH}-\text{Log}\frac{\left[B\right]}{\left[{\text{BH}}^{+}\right]}$$

### 2.4 Nanofiltration separation

In order to ensure that the separation performance of the membranes was not changed during filtration experiments, step 1, NF membrane was washed with deionized water to decrease membrane pollution and to maintain membrane flux. The flux was controlled by transmembrane pressure which was adjusted by the power and flow velocity valve of peristaltic pump. Step 2, remaining water was pumped from the NF apparatus to decrease dilution effect. Step 3, as the membrane flux dropped by 10%, repeat step 1.

During the experiment, the channel of feed solution, filtrate solution, and rejected solution were placed in the same container. In order to improve NF membrane pollution, synephrine extract solution was pretreated by a polyether sulfone microfiltration membrane with a pore diameter of 0.45 μm and a filtration area of 0.3 m^2^. Before sampling analysis, membrane module was pressurized at the test pressure for minimum 2 hr to reach the steady‐state conditions and the adsorption–desorption equilibrium between solutes and membrane. The concentrations of the feed and permeate were analyzed with Agilent 1100. The rejection was calculated according to Equation (1).

(2) $$\text{Rejection}\left(\%\right)=\left(1-\frac{C_{p}}{C_{o}}\right)\times 100\%$$

where *C_o_* and *C_p_* are the solute concentrations in feed and permeate solution. Each measurement was performed in triplicate.

The initial concentration of synephrine extract solution was 200, 150, 100, 50, and 10 μg/ml for transmembrane pressure 0.2, 0.4, 0.6, 0.8, 1.0, and 1.2 MPa, respectively. The pH of synephrine extract solution was changed according to pK_a_, so that synephrine can be divided into three forms (ionic state, molecular state, and mixed state).

### 2.5 Nanofiltration mass transfer model

Based on the solution–diffusion effect (Paul, 2004; Weng et al., 2016; Wijmans & Baker, 1995), it is assumed that in a NF process, the solvent and solute dissolve in the liquid–membrane interface after approaching to the film material, and then, the absorbed molecules transport through the membrane as a consequence of concentration gradient and pressure difference. The equations of the solution–diffusion model can be written as:

(3) $$J_{V}=L_{p}\left(p-\Delta \pi \right),$$

(4) $$N_{A}=\frac{\text{DK}}{\delta }\cdot \left(C_{m}-C_{P}\right)$$

where *J_v_* (m/s) is the solute volume flux; *L_p_* (L/m^2^ hr Pa) is the hydraulic permeability coefficient; *p* (Pa) is operating pressure; Δ*π* (Pa) is the osmotic pressure difference across the membrane; *K* is the solute partition coefficient; *δ* (cm) is the effective thickness of a film; DK*/δ* (cm/s) is the solute transport parameter; *N_A_* (mol/cm^2^ s) is the solute flux; *C_m_* (mol/L) is the total concentration of solute in the liquid–membrane surface; *and C_p_* (mol/L) is the total concentration of solute in the permeate.

The rejections of solute can be divided into the observed rejection (*R_o_*) and the real rejection (*R_r_*), *R_o_* is experimentally measurable interception, which can be calculated by Equation (5). *R_r_* is the real rejection of components, which is difficult to calculate from experimental data due to the value of *C_m_* is hard to be determined directly. *R_o_* is used for determining the membrane parameters.

(5) $$R_{o}=\frac{C_{o}-C_{p}}{C_{o}}$$

(6) $$R_{r}=\frac{C_{m}-C_{p}}{C_{m}}$$

Based on the solution–diffusion model and Equation (3)‐Equation (6), the relationship between *R_o_* and *k* can be expressed as:

(7) $$\ln\left[\left(1-R_{o}\right)\cdot J_{V}/R_{o}\right]=\ln\left[\text{DK}/\delta \right]+\frac{J_{v}}{k}$$

Equation (7) shows that the ln[(1‐*R_o_*)·*J_v_/R_o_*] is linearly related to *J_v_*, with 1/*k* as the slope and ln[DK/δ] as the intercept. Based on different operating pressure and concentration, the prediction model of the rejection is constructed by fitting *k*, ln[DK/*δ*], and the original concentration of synephrine (*C_o_*) at 25°C.

### 2.6 HPLC conditions

Analysis for synephrine was carried out on a Hanbon C_18_ column (250 × 4.6 mm, 5.0 μm). The binary mobile phase consisted of potassium phosphate monobasic (0.6 g potassium phosphate monobasic, 1.0 g sodium dodecyl benzene sulfonate, and 1 ml acetic acid were added to 1,000 ml deionized water) and methanol (50:50, v/v) at a rate of 1.0 ml/min. The detection wavelength was set to 275 nm. The column temperature was sustained at 30°C, and the injection volume was 10 μl.

### 2.7 Linear relationship

0.05, 0.10, 0.20, 0.50, 1.00, and 2.00 ml synephrine reference solution were, respectively, added to 10 ml methanol, and these samples were detected by Agilent 1100 HPLC. The calibration curve was operated by plotting the peak areas (*Y*) versus the concentration of synephrine (*X*), the linear regression equation of which was *Y* = 5.32 *X* + 5.57 (*R* ^2^ = 0.999 2). The calibration curve showed excellent linearity over the concentration ranges of 5.1–204.0 μg/ml for synephrine.

### 2.8 Separation model verification

According to the pK_a_ of synephrine, *Citrus aurantium* extract, of which the concentration of synephrine was detected by HPLC, was, respectively, adjusted to the completely ionic state, the totally molecular state, and the mixed state. The solutions were microfiltered through a 0.45‐μm‐pore size membrane. The *R_o_* value of synephrine at the different transmembrane pressure, respectively, equaled to 0.2, 0.4, 0.6, 0.8, 1.0, and 1.2 MPa, and the predicted one of model was compared with verify the practicability of the mass transfer model.

### 2.9 Evaluation of lipid‐lowering activity

Before the experiment, 50 *SD* rats were given basic feed for 3 days in the experimental environment. Random grouping after adaptation: The blank group continued to feed on the standard food, and 40 *SD* rats in the high‐fat model group were fed high‐fat diet. The rats were weighed every day, and the high‐fat model was considered to be successful when the weight of the rats in the high‐fat model group was at least 20% higher than that in the blank group.

The high‐fat model group was randomly divided into four groups, 10 *SD* rats for each group, namely the high‐fat model group, the positive drug control group, and the *Citrus aurantium* extracts and concentrate group. Moreover, the positive drug control group: orlistat 60 mg/(kg d), the high‐fat model group was administrated equivalent distilled water, *Citrus aurantium* extracts group: 0.5 ml/(kg d), and *Citrus aurantium* concentrate group: 0.5 ml/(kg d).

After modeling, continuously administrate drug by drenching for 4 weeks, then put them to anesthesia, and get the orbital venous blood to do the test of TC, TG, HDL‐C, and LDL‐C content (Wang et al., 2019).

## 3 RESULTS AND DISCUSSION

### 3.1 pK_a_ of synephrine with different concentration

The pK_a_ of synephrine is between 8.25 and 8.84 with the concentration of synephrine ranges from 10 μg/ml to 200 μg/ml, respectively. In order to study the influence of pH of solution on mass transfer coefficient, the synephrine extract solution was changed to pH 4.0 (ionic state), pH 10 (molecular state), and pH 8.5 (mixed state).

### 3.2 Flux of nanofiltration

**Figure 1:**
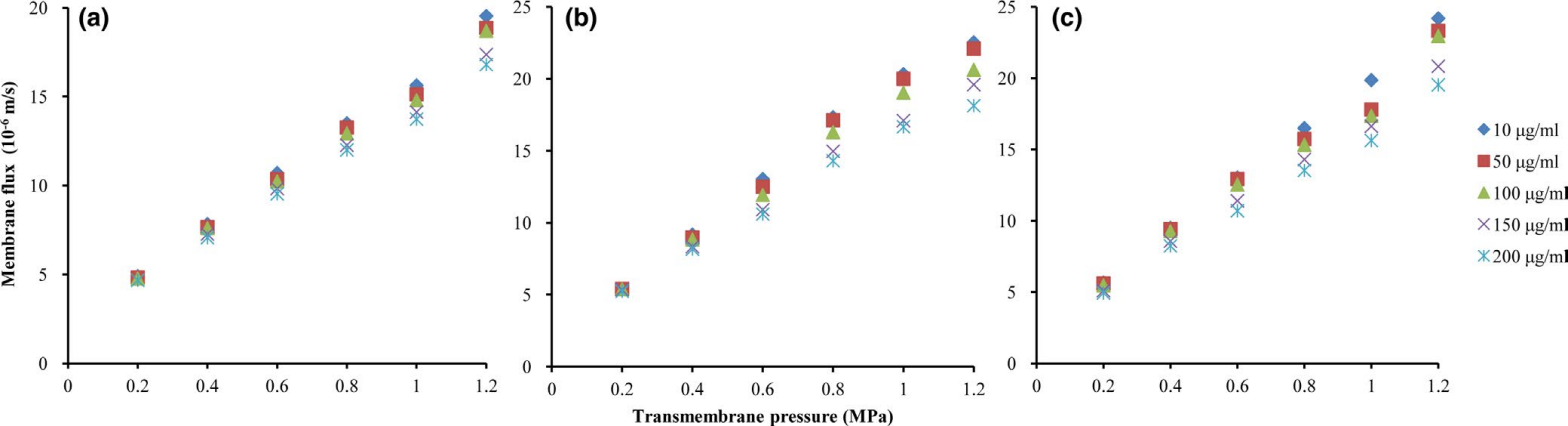
Influence of initial synephrine feed concentration and transmembrane pressure on the nanofiltration flux, (a) ionic state, (b) mixed state, and (c) molecular state

The efficiency of NF separation is usually measured by permeate flux. Permeate flux presents a good linear with pressure by analyzing flux with different operation pressure and original concentration of synephrine (Figure 1), and Equation (3) shows linear positive correlation between transmembrane pressure and permeate flux.

Furthermore, the increasing of original concentration of synephrine leads to a slight drop in permeate flux, which is mainly due to the rise of solution viscosity caused by higher solute concentration. The difference of permeate flux in different concentration shows more obvious when the operating pressure rises, which is owing to the fouling of membrane caused by concentration polarization (Arend et al., 2017).

What's more, pH has some effects on permeate flux as shown in Figure 1. With the elevated pH value of the solution, the flux shows an increasing tendency, especially in pH 4.0. It was supposed that the electrostatic repulsion between ionic state of synephrine and the charged membrane surface was stronger than that of molecular state, so it was difficult for ionic synephrine to get through the membrane surface, and thus, the flux of ionic synephrine is lower than molecular synephrine.

### 3.3 Constructing mass transfer model

#### 3.3.1 Molecular state

**Figure 2:**
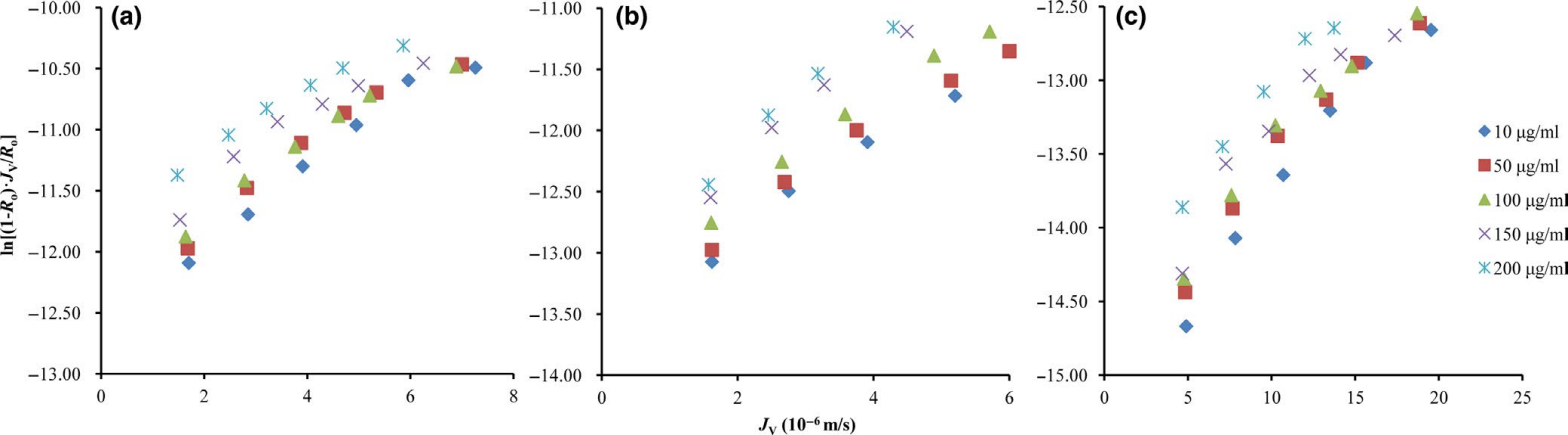
The correlation of ln[(1‐*R_o_*)·*J*_v_/*R_o_*] and *J*_V_ at different initial synephrine concentrations, (a) molecular state, (b) mixed state, and (c) ionic state

By fitting linear relativity between ln[(1‐*R_o_*)·*J*_v_/*R_o_*] and *J_v_* under different operating pressure and concentration (*C_o_*) of synephrine (Figure 2a), the values of *k* and ln[DK*/δ*] are shown in Table 1, which were calculated by linear equation, and these dates show that with the increase in *C_o_*, the *k* increases, and this result agreed well with solution–diffusion model.

**Table 1:** The values of *k* and ln[DK/*δ*] at different initial synephrine concentrations

*C*_o _(10^−7^ mol/L)	*k *(10^−6^ m/s)			ln[DK/*δ*]		
	pH 10.0	pH 8.5	pH 4.0	pH 10.0	pH 8.5	pH 4.0
0.60	10.99	9.05	7.12	−12.54	−13.58	−15.21
2.99	11.63	9.42	7.77	−12.31	−13.45	−14.89
5.98	12.47	9.72	7.94	−12.20	−13.28	−14.77
8.97	12.76	9.88	8.12	−11.97	−13.17	−14.63
11.96	13.33	10.16	8.44	−11.68	−13.10	−14.29

#### 3.3.2 Mixed state

The slope of correlative curves between ln[(1‐*R_o_*)·*J_v_*/*R_o_*] and *J_v_* increased as pH rose from 8.5 to pH 10.0 (Figure 2b), but the mass transfer coefficient of synephrine declined. Both of the molecular and ionic synephrine coexisted in the synephrine extract solution, the molecular synephrine had priority to attach to and dissolve in the membrane surface and then get through the membrane pore by transmembrane pressure. However, the ionic synephrine is difficult to pass the membrane pore due to the charge effect, so the *k* values tend to decrease (Table 1), which also can be used to determine the extent of the mass transfer process impact of the charge effect. Meanwhile, the higher concentration of synephrine leads to the higher *k*, which indicates that the NF process depended not only on solution–diffusion model, but also on the charge effect.

#### 3.3.3 Ionic state

Ionic synephrine was difficult to get through the appearance of NF membrane because there was charge repulsion between synephrine and NF membrane. The *k* values of ionic synephrine dramatically decline compared with the molecular state data (Table 1), in that case, the charge effect was the leading factor in the process of NF separation, while the degree of rejection improved obviously contrasted to other solution environment, and the rejection was over 90% when it was at low concentrations. Furthermore, the value of ln[DK/*δ*] is relevant to the state of synephrine rather than the initial concentration of it. In addition, the value of ln[DK/*δ*] is higher in molecular state than in ionic state (Figure 2c).

### 3.4 Verifying mass transfer model

The results showed that *k* performed correlation function with concentration by fitting the data of them at different pH conditions (4.0, 8.5, and 10.0), respectively. The data of ln[DK/*δ*] and the relevant equation of *k* and *C_o_* are presented in Table 2, and then, the data of *J_v_* and *R_o_* can be calculated by Equation (7).

**Table 2:** The correlation of *k* and *C*_o_ at different pH

pH	Equation	*R* ^2^
4.0	$k=11.18\;C_{o}^{0.06}$	0.935
8.5	$k=9.16\;C_{o}^{0.04}$	0.949
10.0	$k=7.30\;C_{o}^{0.05}$	0.974

**Figure 3:**
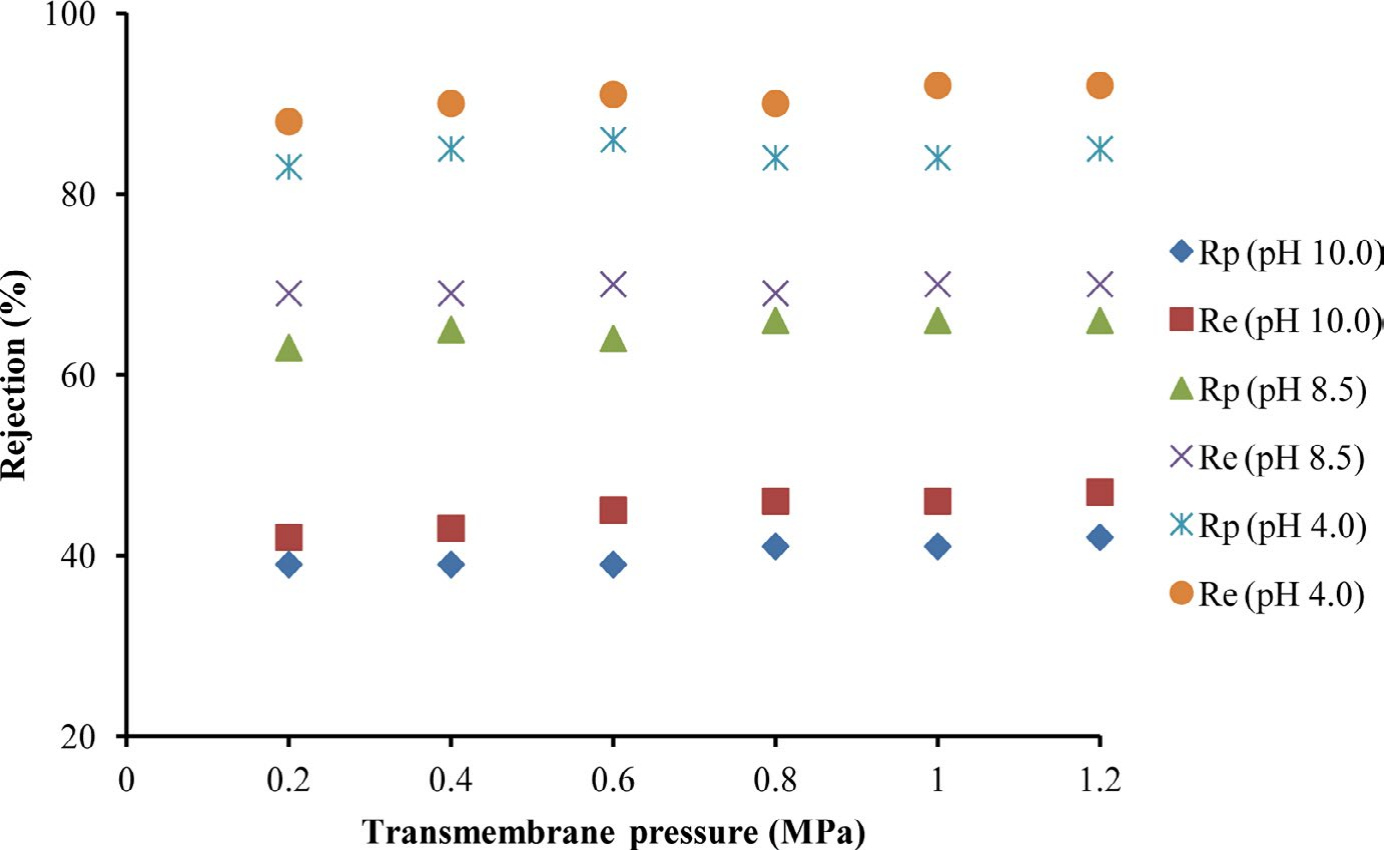
Comparison of the rejections between the experimental values and the predicted values of synephrine with different pH (*R_e_*: the experimental rejection; *R_p_*: the predicted rejection

The concentration of synephrine in *Citrus aurantium* extract was 95.5 mg/ml, detected by HPLC, so that the *R_o_* values under different pH conditions were figured out. Results showed that experimental value matched prediction value, and mass transfer model could be available to predict and evaluate the NF separation characteristics (Figure 3). Some components of *Citrus aurantium* extract competed with each other when they transported through membrane surface, which can be the reason why experimental value was a little higher than prediction value. Besides, flavonoids, such as naringin and neohesperidin, in aqueous extract of *Citrus aurantium*, may combine with synephrine in the form of complex structures, which resulted in the increase of molecular weight as well as *R_o_*. Meanwhile, the growing trend was more obvious for the rejection of experimental value to increase as the operating pressure rose. It was speculated that concentration polarization, caused by high operating pressure, resulted in the rise of *R_o_*.

### 3.5 Effect of synephrine samples on serum lipids of obese rat

**Figure 4:**
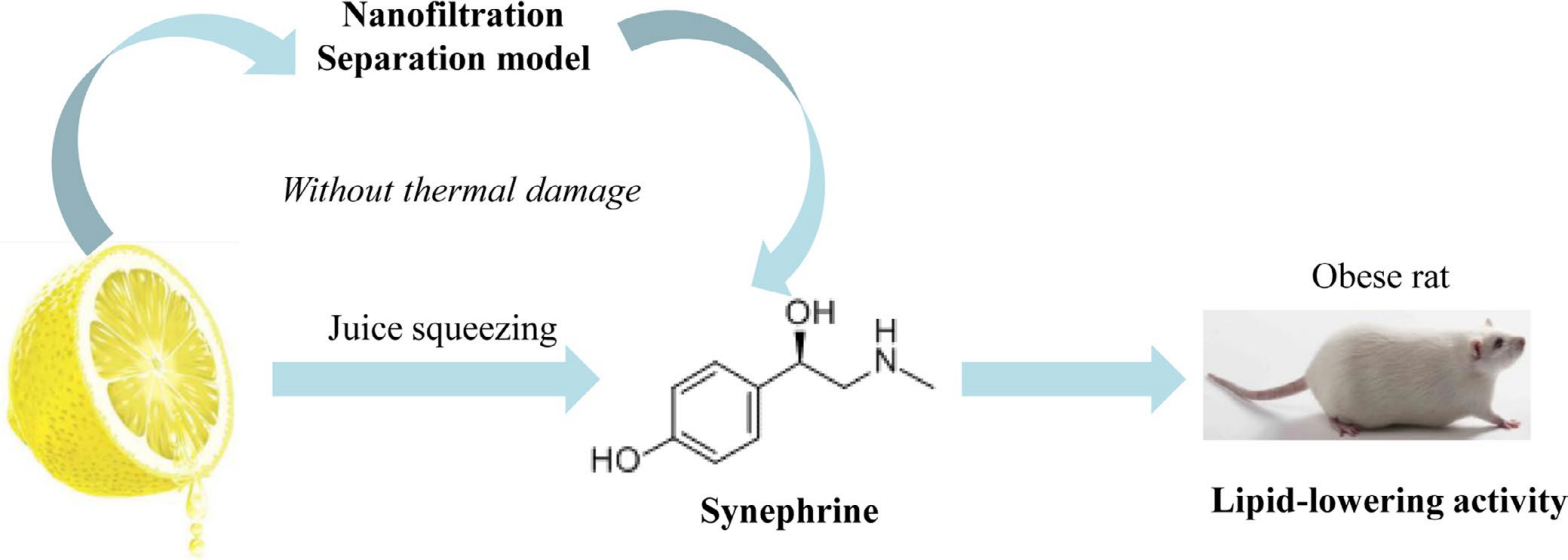
Separation synephrine from *Citrus aurantium* L. (Rutaceae) by nanofiltration technology without thermal damage

Compared with the control group (Table 3), the level of TC, TG, and LDL‐C in serum of the high‐fat model group was significantly increased, but HDL‐C was markedly lower than that in the control group (*p* < .01). Compared with the *Citrus aurantium* extracts group, the level of TC, TG, and LDL‐C in serum of the *Citrus aurantium* concentrate group was significantly decreased (*p* < .01), and HDL‐C content was significantly increased (*p* < .01). After NF concentrating, the concentration of synephrine was effectively increased and the lipid‐lowering activity was also enhanced (Figure 4).

**Table 3:** Effect of synephrine samples on serum lipids of obese rat ($\bar{X}$ ± *S*, *n* = 10) mmol/L

Group	TC	TG	HDL‐C	LDL‐C
Control	1.89 ± 0.11	0.84 ± 0.03	1.32 ± 0.09	0.67 ± 0.07
High fat	2.97 ± 0.32*	1.33 ± 0.08*	0.82 ± 0.02*	1.25 ± 0.17*
Positive control	2.28 ± 0.39	0.90 ± 0.05	1.14 ± 0.08	0.81 ± 0.04
*Citrus aurantium* extracts	2.61 ± 0.45	1.18 ± 0.07	0.93 ± 0.05	1.16 ± 0.06
*Citrus aurantium* concentrate	2.25 ± 0.33**	0.88 ± 0.06**	1.05 ± 0.02**	0.84 ± 0.03**

Note

Compare with a blank group:

* *p* < .01; compare with the *Citrus aurantium* extracts group:

** *p* < .01.

## 4 CONCLUSION

Synephrine was separated and refined by NF technology at room temperature. In the separation process, synephrine molecules dissolved in the membrane surface and then transferred through membrane pore. The NF membrane surface carries negative charges, and as the pH of solution changes, ionic synephrine was difficult to attach to the membrane surface and then make synephrine hard to pass through membrane pores. Under the same concentration, the mass transfer coefficient was directly related to the pH.

In the process of fitting data and constructing separation model, membrane flux was directly related to the accuracy of model prediction, but the membrane fouling was caused by water‐insoluble particles, such as protein, fiber, and other polysaccharide. To ensure accuracy of model prediction, the natural juice of *Citrus aurantium* needed to be pretreated by microfiltration membrane to improve its clarity before NF separation.

By taking solution–diffusion effect, charge effect, and the solute state into account, the mass transfer predicted model of synephrine was established to predict the rejection of synephrine effectively. It initially solved the problem of promotion on the industrialization of NF, which was caused by the unclear separation mechanism of NF for food chemistry.

In this paper, NF separation is an effective technique for the concentration of synephrine from *Citrus aurantium* and the lipid‐lowering activity was also enhanced. In addition, the NF technology can not only improve the technical level and products quality of raw food materials, but also be beneficial to the rationalization of nature plants resources.

## CONFLICT OF INTEREST

The authors have declared no conflict of interest.

## ETHICAL APPROVAL

This study involved animal testing and was approved by the Institutional Review Board of the Nanjing University of Chinese Medicine. The protocol and procedures employed were ethically reviewed and approved by the same organ.
